# Cyclic AMP is a key regulator of M1 to M2a phenotypic conversion of microglia in the presence of Th2 cytokines

**DOI:** 10.1186/s12974-015-0463-9

**Published:** 2016-01-13

**Authors:** Mousumi Ghosh, Yong Xu, Damien D. Pearse

**Affiliations:** The Miami Project to Cure Paralysis, University of Miami Miller School of Medicine, Miami, FL 33136 USA; Department of Neurological Surgery, University of Miami Miller School of Medicine, Miami, FL 33136 USA; The Neuroscience Program, University of Miami Miller School of Medicine, Miami, FL 33136 USA; The Interdisciplinary Stem Cell Institute, University of Miami Miller School of Medicine, Miami, FL 33136 USA

**Keywords:** Interleukin, Alternative activation, Repair, Inflammation, Innate immunity, M1, M2, Arginase, Inos, Phenotype

## Abstract

**Background:**

Microglia and macrophages play a central role in neuroinflammation. Pro-inflammatory cytokines trigger their conversion to a classically activated (M1) phenotype, sustaining inflammation and producing a cytotoxic environment. Conversely, anti-inflammatory cytokines polarize the cells towards an alternatively activated (M2), tissue reparative phenotype. Elucidation of the signal transduction pathways involved in M1 to M2 phenotypic conversion may provide insight into how the innate immune response can be harnessed during distinct phases of disease or injury to mediate neuroprotection and neurorepair.

**Methods:**

Microglial cells (cell line and primary) were subjected to combined cyclic adenosine monophosphate (cyclic AMP) and IL-4, or either alone, in the presence of pro-inflammatory mediators, lipopolysaccharide (LPS), or tumor necrosis factor-α (TNF-α). Their effects on the expression of characteristic markers for M1 and M2 microglia were assessed. Similarly, the M1 and M2 phenotypes of microglia and macrophages within the lesion site were then evaluated following a contusive spinal cord injury (SCI) to the thoracic (T8) spinal cord of rats and mice when the agents were administered systemically.

**Results:**

It was demonstrated that cyclic AMP functions synergistically with IL-4 to promote M1 to M2 conversion of microglia in culture. The combination of cyclic AMP and IL-4, but neither alone, induced an Arg-1^+^/iNOS^−^cell phenotype with concomitant expression of other M2-specific markers including TG2 and RELM-α. M2-converted microglia showed ameliorated production of pro-inflammatory cytokines (TNF-α and IP-10) and reactive oxygen species, with no alteration in phagocytic properties. M2a conversion required protein kinase A (PKA), but not the exchange protein directly activated by cyclic AMP (EPAC). Systemic delivery of cyclic AMP and IL-4 after experimental SCI also promoted a significant M1 to M2a phenotypic change in microglia and macrophage population dynamics in the lesion.

**Conclusions:**

Using primary microglia, microglial cell lines, and experimental models of CNS injury, we demonstrate that cyclic AMP levels are a critical determinant in M1–M2 polarization. High levels of cyclic AMP promoted an Arg-1^+^ M2a phenotype when microglia were activated with pro-inflammatory stimuli and Th2 cytokines. Th2 cytokines or cyclic AMP independently did not promote these changes. Phenotypic conversion of microglia provides a powerful new therapeutic approach for altering the balance of cytotoxic to reparative microglia in a diversity of neurological diseases and injury.

## Background

CNS resident microglia and infiltrating macrophages play a pivotal role in the acute inflammatory response to neurological injury and disease [1], altering the lesion environment to either a cytotoxic milieu characterized by ongoing tissue damage and cell death [2] or a reparative environment, defined by extracellular matrix remodeling, angiogenesis, and axonal regrowth [3]. The functions of these cells and their immunophenotypical characteristics are governed by a variety of signals produced both systemically and at the site of tissue injury, which drive them from a resting state to either a “classically activated,” pro-inflammatory M1 phenotype or an “alternatively activated,” reparative M2 phenotype [4]. Activators of the M1 phenotype, tumor necrosis factor-α (TNF-α), and interferon-γ (IFN-γ), induce microglia to produce pro-inflammatory cytokines, oxidative metabolites, and proteases [5], which although important for microbial defense [6], exacerbate the extent of the initial neural injury and associated dysfunction [7]. Markers of the M1 phenotype include inducible nitric oxide (iNOS), cyclo-oxygenase-2 (COX-2), and cell surface markers such as CD16, CD86, and MHC II. Conversely, the M2 phenotype results from microglial activation in the presence of anti-inflammatory cytokines, including interleukin (IL)-4, IL-10, IL-13, and tumor growth factor-β (TGF-β) [8]. M2 microglia produce a variety of growth factors including vascular endothelial growth factor (VEGF), brain-derived neurotrophic factor (BDNF), and platelet-derived growth factor (PDGF), which are important for promoting angiogenesis, oligodendrocyte maturation, axonal regrowth, and remyelination repair that is conducive to wound healing [9]. The M2a phenotype, a subset of the alternatively activated state of microglia or macrophages, is characterized by the markers Arginase-1 (Arg-1), transglutaminase-2, RELM-α, and YM1 [8, 9].

Though studies have suggested that the presence of Th2 cytokines alone are sufficient for converting a pro-inflammatory, activated M1 form to an M2a phenotype, a persistent M1–M2a conversion using this approach has not been achievable in vivo; macrophages that have been stimulated towards this alternatively activated phenotype by Th2 cytokines alone are unable to retain their immunophenotypical and functional properties when placed within the injured or diseased CNS milieu, reverting instead to the M1 phenotype [10]. In the current study, we have demonstrated that cyclic adenosine monophosphate (cyclic AMP) levels are a critical determinant in pronounced M1 to M2a phenotype conversion when microglial cells are in the presence of pro- and anti-inflammatory stimuli. The elevation of cyclic AMP in microglia or macrophages, through the use of adenylyl cyclase (AC) activators, phosphodiesterase (PDE) inhibitors, synthetic cyclic AMP analogs, or β-adrenergic receptor agonists, inhibits the production of pro-inflammatory genes [11] that are controlled by the master immune regulator, transcription factor nuclear factor kappa B (NF-κB; [12]). We suggest that the role of cyclic AMP signaling is to suppress the M1 phenotype, largely by inhibiting NF-κB, thus potentiating the alternative activation of microglia into an augmented, M2a phenotypic state synergistically in the presence of Th2 cytokines.

## Methods

### Reagents

Cytokines were purchased from PeproTech (Rocky Hills, NJ). Cyclic AMP analogs were obtained from the BIOLOG Life Science Institute (Bremen, Germany) except dioctynyl cyclic AMP, which was obtained from Santa Cruz Biotechnology Inc. (Dallas, TX). Lipopolysaccharide (LPS) was purchased from Sigma-Aldrich (St. Louis, MO).

### BV2 microglial cell culture

The immortalized BV2 microglial cell line employed for in vitro experiments was derived from the C57BL/6 mouse [13] and exhibits phenotypic and functional properties of primary microglia [14]. BV2 microglia were cultured at 37 °C, 5 % CO_2_ in Dulbecco’s modified Eagle’s medium (DMEM; Gibco, Life Technologies Corporation, Grand Island, NY) supplemented with 10 % heat-inactivated fetal bovine serum (FBS; HyClone, Logan, UT) and 100 units/mL each of penicillin and streptomycin (Sigma-Aldrich). Prior to experimental use, BV2 microglia were seeded on either six-well culture plates (5 × 10^4^ cells/well for biochemistry experiments) or eight-well chamber slides (5 × 10^3^ cells/well for immunocytochemistry) and grown to 70–80 % for experimental use. BV2 microglia were treated with various cytokines or pharmacological agents based on the experimental schema described below.

### Primary microglial cell culture

Primary cultures of microglia were prepared from the cerebral cortex of neonatal (P1) wild type C57Bl/6J mice (JAX® Mice and Services, Bar Harbor, ME) using previously published methods [15]. Briefly, the cerebral cortices were dissected and cut into 2-mm small pieces. Tissue was suspended in cold DMEM (Gibco, Life Technologies Corporation) and triturated using a P-1000 plastic tip. The resulting cell suspension was passed through a 100-μm cell strainer and then centrifuged at ×1000 rpm for 10 min. The supernatant was removed and the pellet re-suspended in DMEM containing 10 % FBS and 10 % horse serum (Gibco, Life Technologies Corporation). The cell suspension was plated on polylysine (PLL)-coated T75 flasks and incubated at 37 °C for 7 to 10 days. Culture flasks were then placed on an orbital shaker at X230 rpm for 3 h, and the media, containing detached microglia, was harvested. Following centrifugation for 10 min at ×1000 rpm, a pellet containing the microglial cell fraction was acquired and re-suspended in the same media as described above. Highly pure microglial cell cultures were obtained using this method (>95 % as assessed using immunocytochemistry for CD11b (AbD Serotec; Raleigh, NC) and iba1 (Wako Pure Chemicals; Richmond, VA)). Primary microglia were diluted to the desired cell concentration and plated for at least 24 h before experimental use.

### M1 to M2 phenotypic conversion

M1 activation was achieved with LPS (100 ng/ml) or TNF-α (10 ng/mL). For M1 to M2a phenotype conversion, IL-4 (10 ng/mL) was used in combination with cyclic AMP analogs (dibutyryl cyclic AMP; (1 mM), 6-Phe-cyclic AMP; (100 μM), CPT-2′O cyclic AMP; (100 μM)) beginning at 15 min prior to M1 activation. Cell supernatants were analyzed for cytokine levels, cell lysates prepared, or the cells fixed with 4 % paraformaldehyde 24 h later to measure the expression of M1 and M2a phenotype markers. All data are representative of at least three independent experiments.

### Assessment of phagocytic function

Phagocytic function was evaluated based on the uptake of phycoerythrin (PE)-conjugated latex beads (Phagocytosis Assay Kit; Cayman Chemical, Ann Arbor, MI) as per the manufacturer’s protocol. Percent phagocytic cells was quantified in relation to the total number of Hoechst-positive nuclei in four random fields per treatment well and averaged across five independent experiments by fluorescence microscopy.

### Western blotting

Immunoblotting was performed on culture supernatants, total cell lysates, or spinal cord (T7–9) tissue homogenates to quantify the expression of M1 and M2a markers according to previously published methods [15]. Nitrocellulose membranes (Bio-Rad Laboratories) were probed with specific primary antibodies; Arg-1 (1:2,000; GeneTex Inc., Irvine, CA), iNOS (1:1,000; Cell Signaling Technology, Boston, MA), TNF-α (1:500; Life Technologies Corp.), and β-actin (Sigma-Aldrich). The optical density of the bands (arbitrary units) was measured with an imaging densitometer (Bio-Rad Laboratories) and normalized to β-actin levels. The data represents values from three independent experiments.

### ELISA

Levels of the pro-inflammatory cytokines in culture supernatants was quantified using mouse TNF-α, IP-10, and IL-1β enzyme-linked immunosorbent assays (ELISA; Pepro Tech, Rocky Hill, NJ) according to the manufacturer's protocols. Values were obtained as picogram per milliliter of the culture supernatant and expressed as absolute values and compared between each of the treatment conditions. The data presented are the mean ± SEM of four independent experiments.

### Determination of reactive oxygen species

The presence of reactive oxygen species (ROS) was measured using the non-fluorescent dye 2′,7′-dichlorofluorescein diacetate (DCFH-DA; Molecular Probes, Eugene, OR) as per the manufacturer’s instructions. DCF fluorescence measurements were made in 96-well plates using a SpectraMax 5 microplate reader at 485 nm for excitation and 530 nm for emission. The data obtained were representative of three independently conducted experiments.

### Nitrite assay

Microglia nitrite production was assessed by measuring total nitrite concentration, a stable oxidation product of NO by the Griess reaction. Briefly, culture supernatants (50 μl) were mixed with an equal volume of Griess reagent (Life Technologies Corp.) in 96-well plates for 10 min at room temperature in the dark, and the absorbance at 570 nm was determined using a SpectraMax 5 microplate reader. Sodium nitrite at concentrations of 0 to 100 μM was used as a standard. Each value indicates the mean ± SEM and is representative of results obtained from three independent experiments.

### Immunocytochemistry

M1 and M2 marker expression in microglia was examined using immunocytochemistry (ICC) according to previously published methods [15]. Fixed cells were probed with primary antibodies for the M1 phenotype, iNOS (1:200; BD Transduction Laboratories; Franklin Lakes, NJ), and COX-2 (1:200; Thermo Scientific, Rockford, IL) or the M2 phenotype, Arg-1 (1:200; GeneTex, Irvine, CA), RELMα/Fizz1 (1:100; PeproTech, Rocky Hill, NJ), or transglutaminase-2 (1:100; Thermo Fischer Scientific). In addition, the microglial cell markers anti-iba1 (1:5,000; Wako Pure Chemical Industries, Ltd., Tokyo, Japan) and CD68/ED1 (Macrosialin; 1:200; AbD Serotec) were used. For visualization, the secondary antibodies, goat anti-mouse Alexa-594 or anti-rabbit Alexa-594 (1:200, Life Technologies Corp.) were used along with the nuclear marker Hoechst 33342 (1:1,000; Life Technologies Corp.). The morphology of the cells was demarcated by staining with Phalloidin-488 (1:100; Life Technologies Corp). Images were acquired by sequential scanning of the immunostained cells with an Olympus Fluorescence confocal microscope (Olympus, Fluoview FV 1000). For each treatment condition, three to four randomly selected fields were imaged per well. Images obtained were representative of three independently conducted experiments.

### Imaging and analysis

Confocal images were acquired by sequential scanning of the immunostained cells with an Olympus Fluorescence Microscope (Olympus, FluoView FV1000) at laser lines based on the specific Alexa-fluor secondaries used. In Adobe Photoshop CS6 (Adobe Systems, San Jose, CA) fluorescent images had the same universal adjustments applied: brightness (+50), contrast (+30), and smart sharpen (1.0 pixels).

### Measurement of fluorescent intensity per cell

The per cell immunostaining fluorescence intensity of specific markers was quantified using Image J software (http://imagej.nih.gov/ij/). For each treatment condition, randomly selected cells [10, 11] per well/plate from independent experiments were identified by Hoechst-positive nuclei and imaged. These images were converted to grayscale, and the average per pixel intensity of the signal per cell (arbitrary units (a.u.) was recorded after background subtraction. These measurements were averaged across the cells analyzed per plate and then among group replicates for comparison of signal change across treatments.

### Spinal cord contusion injury

Two models of spinal cord injury (SCI) were used in this study to examine the effects of IL4 and db-cyclic AMP on microglia and macrophage phenotypes in rodents. The first model employed adult Lewis rats (female, 180–200 g, *n* = 3), the second C57BL/6 (female, 25 g) mice (both from Charles River Laboratories International Inc., Wilmington, MA). Animals were housed according to NIH guidelines and The Guide for the Care and Use of Animals. All animal procedures were approved by the University of Miami Institutional Animal Care and Use Committee (IACUC). The rats were subjected to a moderate (25.0 mm) thoracic (T8) spinal cord contusion induced by the MASCIS weight drop device developed at New York University [16] as described previously [17]. For the mouse model (*n* = 3 per group) animals were subjected to a 50 kdyne moderate spinal cord contusion at thoracic (T8) level using the Infinite Horizons (IH) impactor as described previously [18]. Post-operative care was provided as described in Patel et al. [19].

### Administration of M2a converting agents

With the Lewis rat model, IL-4 and db-cyclic AMP were delivered by intravenous (i.v.) administration through a jugular vein catheter prepared as described previously [20]. A mix of db-cyclic AMP (50 mg/kg) and IL-4 (30 μg/kg) in 100 μl of sterile, deionized water was administered as a single bolus injection with a 0.5-ml tuberculin syringe attached to the i.v. catheter over a period of 2 min within 15 min of SCI, followed by a 250-μl physiological saline flush. For the mouse model, the animals received daily, intraperitoneal injections (100 μl) of the agents at the same concentrations beginning at 30 min post-SCI for 7 days until end-point.

### Animal perfusion and tissue extraction

Animals were deeply anesthetized (150 mg/kg ketamine, 10 mg/kg xylazine) and transcardially perfused and the extracted CNS tissue post-fixed and cryoprotected as described elsewhere [21]. The T7–9 spinal cord was cryosectioned at a thickness of 20 μm (coronal) on a Leica CM3050S Cryostat (Leica Microsystems Inc., Buffalo Grove, IL). For biochemistry, animals were deeply anesthetized at 24 h post-SCI and decapitated. A spinal cord segment (5 mm) encompassing the injury epicenter dissected and snap frozen in liquid nitrogen for storage at −80 °C until processed for analysis.

### Immunohistochemistry

Coronal, 20-μm cryosectioned sections of injured spinal cord tissue (400-μm intervals) were immunohistochemically (IHC) stained for specific markers using methods as described elsewhere [20]. For IHC, the Arg-1 antibody (1:200) was combined with anti-CD68 (ED1; 1:200; AbD Serotec), Alexa-594 tagged Isolectin-IB4 (1:100; Life Technologies Corp.) or anti-iba1 (1:5,000; Wako) along with appropriate AlexaFluor conjugated secondary antibodies (1:200; Life Technologies Corp.) and Hoechst 33342 (Sigma-Aldrich).

### Quantification of microglial cells at the lesion site

For quantifying the conversion of microglia and macrophages to M2a, cells displaying double immunoreactivity for Arginase-1 (Alexa-488 secondary antibody) and Alexa-594-tagged Isolectin-IB4 were quantified from projection images obtained using a confocal microscope (Olympus, Fluoview 1000) at ×40 magnification. These images were taken from within the injury epicenter, from the dorsal funiculus region of the spinal cord (*n* = 3 mice per group of IL-4 and db-cyclic AMP-treated and SCI-only controls). Images were converted to 8-bit gray scale separately for each marker using Image J software, and a threshold value was obtained. The relative percent of cells that were displaying the M2a phenotype were analyzed by measuring the fluorescent intensity of the number of cells showing Arginase-1 immunoreactivity and the total number of Isolectin-IB4^+^ cells between the treated and the untreated groups.

### Statistics

Significant differences between groups were ascertained by a one-way analysis of variance (ANOVA) or a *t* test, followed by a Bonferroni post-hoc analysis using Graph Pad Prism 4.0 (Graphpad Software, La Jolla, CA). All data was analyzed at the 95 % confidence interval. Graphed data was expressed as the mean ± standard error of the mean (SEM) according to the number of replicates or independent experiments. Asterisks or hashes included on the graphs indicate statistical differences between the treatment and control condition(s) with significance indicated at ***/^###^*p* < 0.001, **/^##^*p* < 0.01, or */^#^*p* < 0.05.

## Results

### Cyclic AMP and IL-4 synergize to augment expression of M2a phenotypic markers in microglia induced to an M1 form by pro-inflammatory mediators

**Fig. 1:**
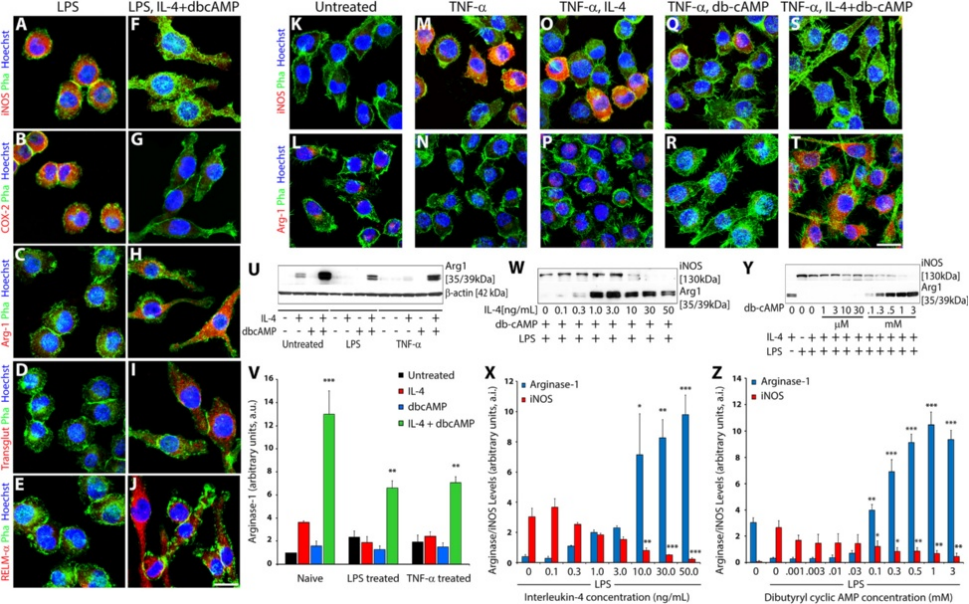
Synergistic action of cyclic AMP and IL-4 in M1 to M2a phenotypic conversion of microglial cells. In LPS (100 ng/ml)-stimulated BV2 microglia, combined treatment with db-cyclic AMP (1 mM) and IL-4 (10 ng/ml) beginning at 15 min prior to M1 activation produced dramatic reductions in the M1 markers (*red*) iNOS (**a**, **f**) and COX-2 (**b**, **g**) at 24 h post-stimulus while concurrently increasing M2a markers (*red*) Arginase-1 (**c**, **h**), transglutaminase-2 (**d**, **i**), and RELM-α (**e**, **j**). *Scale bar* = 12 μm. Similarly, conversion of M0 BV2 microglia (**k**, **l**) to M1 with TNF-α produced robust iNOS expression (**m**) without immunoreactivity for Arginase-1 (**n**). IL-4 (10 ng/ml) alone produced some expressions of Arginase-1 (**p**) but did not alter iNOS production (**o**), while db-cyclic AMP (1 mM) reduced iNOS and induced little Arginase-1 expression in TNF-α-stimulated microglia (**r**). Only the simultaneous addition of IL-4 and db-cyclic AMP to TNF-α-induced M1 microglia produced profound M2a conversion as evidenced by a loss of iNOS (**s**) and strong Arginase-1 expression (**t**). Microglial cells are stained with phalloidin-488 (*green*) to demark the cell morphology, and the nuclei of the cells have been counterstained with Hoechst (*blue*). *Scale bar* = 20 μm. Immunoblotting showed that only the combination of db-cyclic AMP and IL-4 produced a robust induction of Arginase-1 in M0 or M1 microglia (stimulated with either LPS or TNF-α; **u**, **v**). Immunoblotting for Arginase-1 and iNOS in LPS-stimulated BV2 microglia following their exposure to increasing concentrations of IL-4 (fixed db-cyclic AMP concentration, **w**, **x**) or with increasing concentrations of db-cyclic AMP (fixed IL-4 concentration, **y**, **z**) shows the concentrations at which synergistic action is observed in their conversion of microglia from M1 to M2a. Densitometry values for Arginase-1 and iNOS were normalized to β-actin. Statistical significance indicated at ***p < 0.001, **p < 0.01, or *p < 0.05 versus LPS-only-treated controls. Results are shown from three independent culture replicates

BV2 microglia exposed to a pro-inflammatory stimulus, LPS, showed M1 phenotype induction as evidenced by pronounced expression of the characteristic markers iNOS and COX-2 and an absence of the M2a markers, Arginase-1, transglutaminase-2, and RELM-α (Fig. 1a–e). Concurrent treatment of LPS-stimulated BV2 microglia with IL4 and db-cyclic AMP but, neither independently (data not shown), antagonized M1 marker expression while dramatically augmenting an M2a phenotype as demonstrated by robust expression of M2a markers (Fig. 1f–j). M1 to M2a phenotype conversion in BV2 microglia was demonstrated immunocytochemically also with another pro-inflammatory mediator, TNF-α, in which, compared to untreated (Fig. 1k–l) or TNF-α-stimulated controls (Fig. 1m, n), combined IL-4 and db-cyclic AMP induced robust Arginase-1 production and the antagonism of iNOS expression (Fig. 1s, t). Only minor changes in Arginase-1 and iNOS were observed in TNF-α-stimulated BV2 microglia treated with IL-4 (Fig. 1o–p) or db-cyclic AMP (Fig. 1q–r) independently at the doses employed. Similarly, modest expression of transglutaminase-2, and RELM-α were detected with IL-4 treatment, but the presence of db-cyclic AMP dramatically potentiated their expression and the alternatively activated M2a phenotype (data not shown).

The necessity of cyclic AMP for robust M1 to M2 phenotype conversion was further demonstrated biochemically by immunoblotting for the prototypical M2 marker Arginase-1 (Fig. 1u, v). In unstimulated, resting microglia (M0 phenotype), exposure to IL-4 alone, but not db-cyclic AMP, elevated levels of Arginase-1, indicative of a transition to an M2 phenotype. However, as observed with LPS- or TNF-α-induced M1 microglia, exposure of the resting M0 microglia to the combination of IL-4 and db-cyclic AMP showed a much more pronounced expression of Arginase-1 (3.6-fold higher, *p* < 0.001). In M1 activated microglia exposed to either LPS or TNF-α, IL-4 and db-cAMP when given independently at the dose used did not induce robust Arginase-1 compared to concurrent application of IL-4 and db-cyclic AMP (Fig. 1u, v).

To examine the dose-dependent effects of IL-4 and db-cyclic AMP on the phenotype conversion of M1 microglia (iNOS^+^) to M2 phenotype (Arginase-1^+^), immunoblotting studies with titrated concentrations of these agents were performed. When the concentration of db-cyclic AMP was kept constant at 1 mM, IL-4 concentrations at and above 10 ng/mL were required to produce a significant induction of Arginase-1 expression and concomitant reduction in iNOS expression in LPS-stimulated BV2 microglia (Fig. 1w, x). Similarly, maintaining IL-4 at 10 ng/mL demonstrated that db-cyclic AMP concentrations at and beyond 0.1 mM could trigger a significant induction of Arginase-1 along with the parallel inhibition of iNOS expression during the phenotype conversion of M1 microglia to M2 (Fig. 1y, z).

### Cyclic AMP profoundly modifies cytokine secretion in microglia stimulated to the M1 phenotype by LPS or TNF-α

**Fig. 2:**
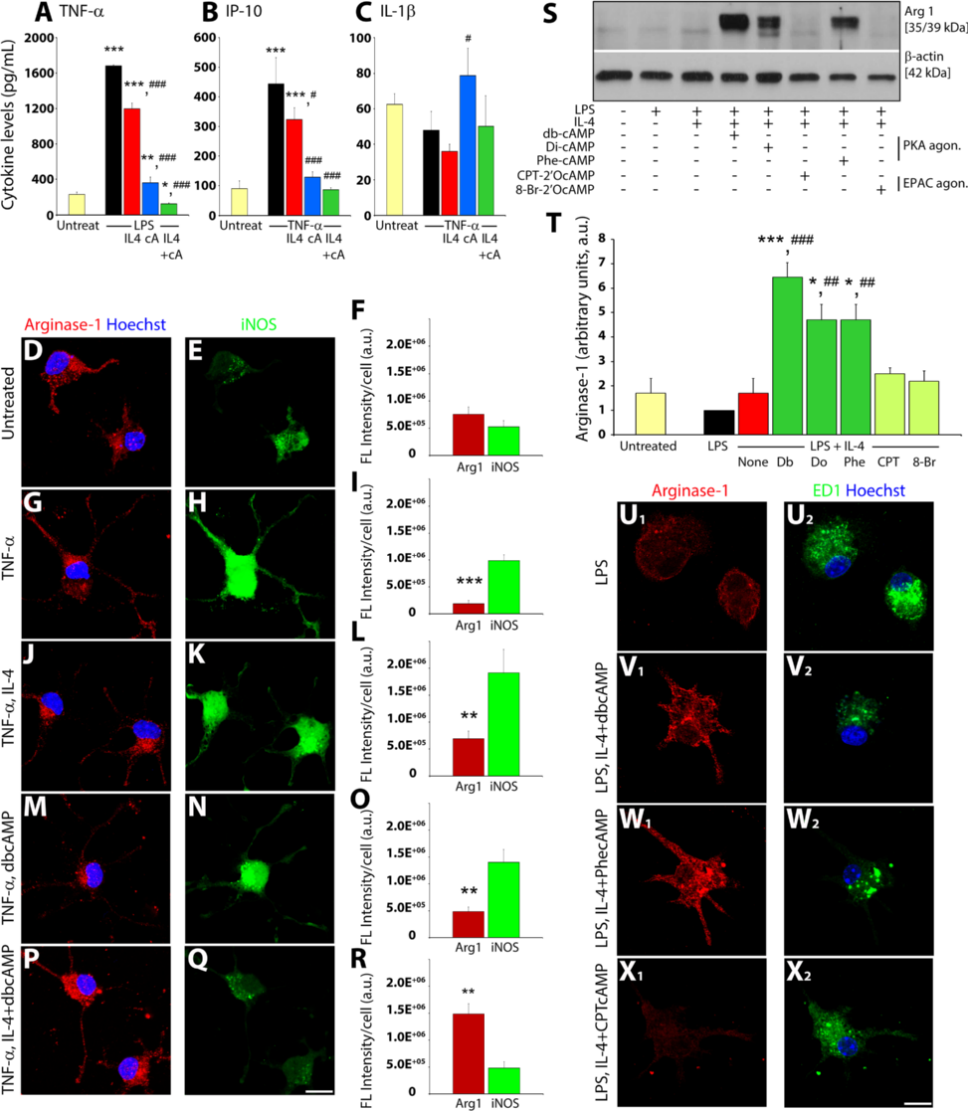
M1 to M2a conversion of microglia alters secretory profiles, occurs at the single cell level, and requires PKA, but not EPAC, signaling in the presence of IL-4. Treatment of M1 (LPS; 100 ng/ml or TNF-α stimulated 10 ng/ml) BV2 microglia with db-cyclic AMP (1 mM) and IL-4 (10 ng/ml) beginning at 15 min prior to activation produced maximal abrogation of cytokines, TNF-α (**a**) and IP-10 (**b**) at 24 h post-stimulus. Levels of IL-1β remained statistically indifferent between M0, M1, and M2a phenotypes (**c**), with only db-cyclic AMP alone producing a significant increase over TNF-α stimulated controls. Examination of primary cortical microglia at the single cell level showed, compared to untreated M0 microglia (**d**, **e**), a significant shift in the ratio of Arginase-1 (*red*) to iNOS (*green*) expression, favoring the latter (**g**–**i**), occurred when the microglia were induced to the M1 form with TNF-α. The exposure of M1 microglia to either IL-4 (**j**–**l**) or db-cyclic AMP (**m**–**o**) independently failed to alter this ratio. In contrast, concurrent use of db-cyclic AMP and IL-4 significantly shifted the ratio of Arginase-1 to iNOS expression to one favoring the former (**p**–**r**), indicative of M1 to M2a phenotypic conversion. Statistical significance indicated at ***p < 0.001 or **p < 0.01 versus iNOS FL intensity/cell. *Scale bar* = 15 μm. **s** Immunoblotting for Arginase-1 in BV2 microglia showed little expression in M0-untreated cells or M1 cells induced with LPS and either untreated or exposed to IL-4. Concurrent delivery of IL-4 with cyclic AMP analogs showed that those that selectively activated PKA (db-cyclic AMP, 1 mM; dioctanoyl-cyclic AMP, 100 μM; phenyl-cyclic AMP, 100 μM), but not EPAC (CPT-2′O methyl-cyclic AMP, 100 μM and 8-bromo-2′O methyl-cyclic AMP, 100 μM), induced robust Arginase-1 expression, indicative of M1 to M2a phenotypic conversion. **t** Densitometry values for Arginase-1 in untreated and treatment groups normalized to β-actin. The expression of Arginase-1 (*red*) and iNOS (*green*) in primary microglia treated with LPS alone (u_1_–u_2_) or in conjunction with IL-4 and cyclic AMP analogs that activate PKA and/or EPAC (v_1_–x_2_), shows the importance of PKA in mediating conversion towards an Arg1+/iNOS- phenotype in the presence of IL-4. Statistical significance indicated at ***p < 0.001 or *p < 0.05 versus untreated controls or ^###^ *p* < 0.001 or ^#^ *p* < 0.05 versus LPS-only-treated controls. **c**–**j** Immunocytochemical staining of primary microglia for Arginase-1 (*red*) and macrosialin (ED1; *green*) in control and treated microglia showed robust induction of Arginase-1 with PKA-selective, but not EPAC-selective, cyclic AMP analogs in the presence of LPS and IL-4. Cell nuclei were counterstained with Hoechst (*blue*). *Scale bar* = 15 μm. Results shown from three independent culture replicates

The cytokine secretory profile of untreated or resting (M0), M1 (induced by LPS or TNF-α stimulation), or M2a (converted from M1 with IL-4 and db-cyclic AMP) BV2 microglia was investigated in culture supernatants at 24 h using specific ELISAs for TNF-α, IL-1β, or IP-10. ELISAs for each of these major pro-inflammatory cytokines were performed from three independent experiments. Compared to M0, the LPS-treated form showed a significant increase in the levels of TNF-α (TNF-α, 7.4-fold increase, p < 0.001; Fig. 2a). Similarly, TNF-α stimulation of the cells produced elevated concentrations of IP-10 in the culture supernatant at 24 h following treatment (IP-10, 4.9-fold increase, *p* < 0.001; Fig. 2b). IL-4 addition alone produced a modest, though significant reduction in the levels of TNF-α and IP-10 compared to stimulated controls; however, a major abrogation in the secretion of these cytokines was achieved with db-cyclic AMP or its combination with IL-4 (Fig. 2a, b). In contrast, IL-1β production following a 24-h TNF-α stimulus did not show any significant differences between the M0, M1, or M2a phenotypes, though M1 microglia exposed to db-cyclic AMP alone did show significantly enhanced levels of IL-1β (1.7-fold increase, *p* < 0.05) compared to stimulated controls (Fig. 2c).

### M1 to M2 conversion of microglia by the synergistic action of IL-4 and cyclic AMP can be observed at the single cell level

The synergistic effects of cyclic AMP and IL-4 in regulating expression of Arginase-1 and iNOS was examined within single cells of primary cortical microglia challenged with TNF-α (Fig. 2d–q). While levels of Arginase-1 and iNOS were low in M0 microglia and not significantly different from one another (Fig. 2d–f), upon TNF-α stimulation, a profound increase in iNOS over Arginase-1 was observed in single microglial cells (Fig. 2g–i). The exposure of M1 microglia to either IL-4 (Fig. 2j–l) or db-cyclic AMP (Fig. 2m–o) was not able to reverse the balance of Arginase-1 to iNOS expression that significantly favored the later in single cells. In contrast, the concurrent exposure of M1 microglia to IL-4 and db-cyclic AMP switched the balance of Arginase-1 to iNOS expression to significantly favor Arginase-1 (Fig. 2p–r), indicative of an M1 to M2 phenotype conversion.

### The cyclic AMP-protein kinase A pathway confers M1 to Arginase-1^+^ M2a phenotype conversion in microglia

The main effector pathways downstream of cyclic AMP are coordinated through PKA and the exchange protein directly activated by cyclic AMP (EPAC), which have been shown to regulate often distinct cellular processes in neural cells [22]. To probe the involvement of PKA and EPAC in M2a phenotype conversion, selective cyclic AMP analogs that activate PKA or EPAC were employed in combination with IL-4 in cultures of primary cortical microglial cells. Similar to db-cyclic AMP, the PKA-specific cyclic AMP analogs, dioctanoyl-cyclic AMP, and 6-phenyl-cyclic AMP, but not the EPAC-selective analogs, CPT-2′O methyl-cyclic AMP or 8-bromo-2′O methyl-cyclic AMP, induced a significant increase in Arginase-1 expression when combined with IL-4 treatment in M1 microglia (Fig. 2s–t). These results were then confirmed using immunocytochemistry for Arginase-1 in primary cultures of microglia (Fig. 2u–x). LPS-stimulated primary microglia exhibited little immunoreactivity for Arginase-1 and robust expression of macrosialin (ED-1), a characteristic marker of activated microglia and macrophages (Fig. 2u_1_–u_2_). Strong Arginase-1 immunoreactivity was observed in M1 microglia exposed to IL-4 when used in combination with the PKA activators db-cyclic AMP (Fig. 2v_1_–v_2_) and phenyl-cyclic AMP (Fig. 2w_1_–w_2_), but not with the EPAC-selective cyclic AMP analog CPT-2′O methyl-cyclic AMP (Fig. 2x_1_–x_2_).

### Cyclic AMP restores IL-4 mediated deficits of phagocytic activity in the M2a phenotype

**Fig. 3:**
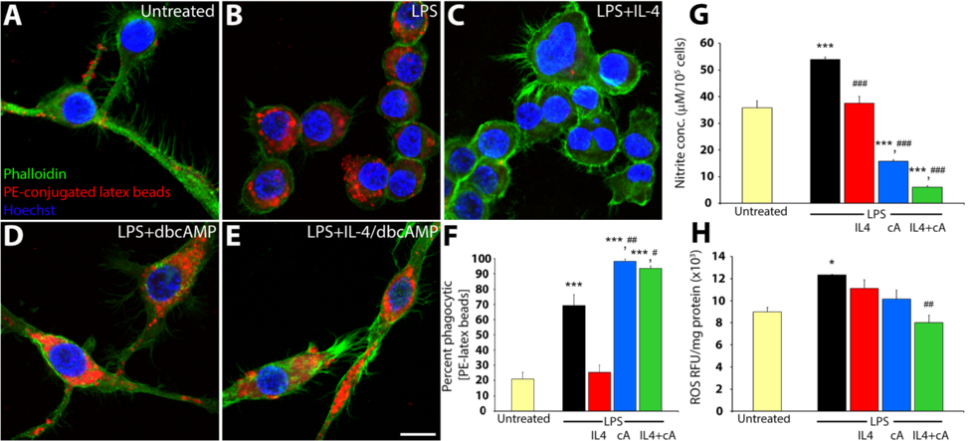
M1 to M2a phenotype conversion of microglia does not perturb their phagocytic function but does reduce the production of reactive species. **a–e** The phagocytic capacity of BV2 microglia was assayed by the ability of the cells to phagocytose phycoerythrin (PE)-conjugated latex beads (*red*) after treatment with LPS (100 ng/ml) in the presence or the absence of IL-4 (10 ng/ml) and db-cyclic AMP (1 mM) added alone or simultaneously 15 min prior to activation and incubated for 24 h post-treatment. Co-staining for nuclei (Hoechst, *blue*) and the cytoplasm (phalloidin-488, *green*) was employed to demark cell morphology. *Scale bar* = 12 μm. **f.** Quantitative analysis of PE-bead uptake. **g**, **h** Measurements of nitrite concentration (**g**) or reactive oxygen species (**h**) in cell lysates from untreated and treated groups were measured at 24 h post-stimulus with conditions inducing the pro-inflammatory or the anti-inflammatory state as mentioned above. Statistical significance indicated at ***p < 0.001 or *p < 0.05 versus untreated controls or ^###^ *p* < 0.001, ^##^ *p* < 0.01, or ^#^ *p* < 0.05 versus LPS-only-treated controls. Results are shown from five independent culture replicates

The phagocytic ability of M2a converted microglia following treatment with LPS and IL4, with or without db-cyclic AMP, was comparatively examined to LPS-stimulated alone (M1) and M0 (no treatment) microglia through quantification of their capacity to phagocytose PE-conjugated latex beads. Compared to untreated cells (Fig. 3a), LPS stimulation of BV2 microglia to an M1 phenotype enhanced their ability to phagocytose PE-conjugated beads (3.3-fold higher than untreated, *p* < 0.001; Fig. 3b, f). In contrast, IL-4 produced a significant impairment in the phagocytic capacity of M1-activated microglia (2.7-fold lower than LPS, *p* < 0.001; ns compared to untreated, Fig. 3c, f). Conversely, the addition of db-cyclic AMP alone significantly increased the phagocytic activity of the M1 phenotype (Fig. 3d) and additionally restored the deficits observed in the phagocytic properties conferred in IL-4 driven M2a microglia (Fig. 3e, f), conferring a phagocytic activity significantly greater than that of LPS stimulation alone (1.4-fold and 1.3-fold higher than LPS, *p* < 0.01 and *p* < 0.05, respectively; Fig. 3f). These results demonstrate that an important role of classically activated M1 microglia, phagocytosis and debris clearance, is retained in the converted M2a phenotype.

### Elevated levels of cyclic AMP combined with IL4 antagonizes the production of reactive oxygen and nitrogen species following microglial activation

We examined whether M2a converted microglia, obtained through the combined exposure of M1 microglia to IL-4 and db-cyclic AMP, produced reactive oxygen (ROS) and/or nitrogen species (RNS), cytotoxic molecules that are associated with the M1 microglial cell phenotype. The ability of M1 microglia to produce RNS, measured by nitrite concentration, was detected based on the Griess reaction. Compared to untreated controls, LPS stimulation of BV2 microglia to the M1 form produced a significant increase in cell nitrite concentration (1.5-fold increase, *p* < 0.001; Fig. 3g). Concurrent use of IL-4 with LPS significantly attenuated this increase (Fig. 3g). The employment of either db-cyclic AMP alone or M2a phenotypic conversion with the combination of IL-4 and db-cyclic AMP, further reduced nitrite concentrations to levels significantly lower than that of untreated controls (2.3-fold and 5.9-fold reduction compared to untreated, *p* < 0.001 for both, respectively; Fig. 3g). Levels of ROS detected using DCFH-DA showed that LPS stimulation of BV2 microglia produced a significant increase in ROS production compared to untreated controls (1.4-fold increase, *p* < 0.05; Fig. 3h). The concomitant use of IL-4 or db-cyclic AMP alone did not significantly reduce the increase in ROS produced by LPS stimulation. Only when db-cyclic AMP and IL-4 were employed in combination to promote M2a phenotype conversion was the LPS-induced generation of ROS in microglia completely perturbed (1.5-fold decrease, *p* < 0.01 compared to LPS stimulation; ns compared to untreated; Fig. 3h).

### Acute systemic co-administration of IL4 and cyclic AMP induces a state of alternative activation in microglia and macrophages after spinal cord injury

**Fig. 4:**
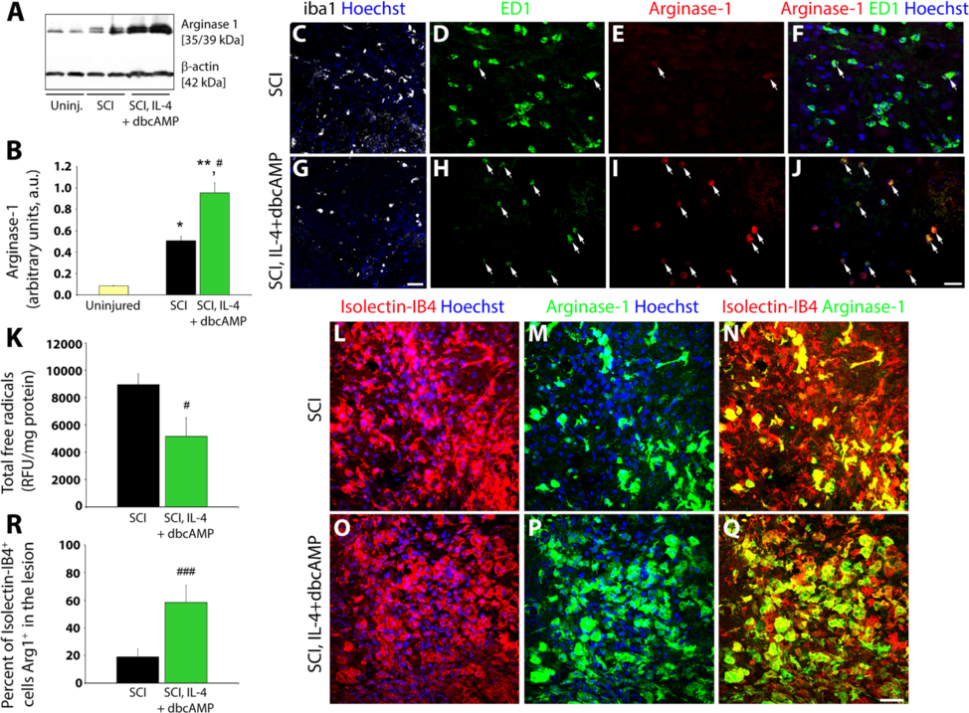
M1 to M2a conversion of microglia with cyclic AMP and IL-4 can be used in vivo after SCI and significantly attenuates the production of tissue-damaging free radicals. **a**, **b** Immunoblotting of tissue homogenates from the injury epicenter at 24 h after contusive SCI in rat for the prototypical M2 marker Arginase-1 shows an increase in expression after SCI that is further potentiated by the co-delivery of db-cyclic AMP (50 mg/kg) and IL-4 (30 μg/Kg) administered <30 min post-injury. Statistical significance indicated at **p < 0.01 or *p < 0.05 versus uninjured controls or ^#^ *p* < 0.05 versus SCI only controls. Compared to SCI controls, the administration of db-cAMP and IL-4 produced a dramatic reduction in the numbers of Iba1^+^ (**c**, **g**) and ED1^+^ (*green*, **d**, **h**) microglia and macrophages within the lesion epicenter at 24 h post-SCI. While few ED1^+^ microglia and macrophages were Arginase-1^+^ (*red*, *white arrows*) in SCI-only controls (**d**, **f**), almost all ED1^+^ microglia and macrophages found in db-cyclic AMP and IL-4-treated animals co-expressed Arginase-1 (**h**, **j**; *white arrows*). The nuclei of the cells have been counterstained with Hoechst (*blue*). *Scale bar* = 20 μm. **k** Compared to SCI only controls, a dramatic reduction in the total level of free radicals within the injury epicenter was observed with the systemic administration of db-cyclic AMP and IL-4. Statistical significance indicated at ^#^ *p* < 0.05 versus SCI only controls. Four animals were used in each group. Following contusive SCI in mouse, compared to injury-only controls (**l**–**n**), daily treatment initiating at <30 min post-SCI induction with IL-4 (30 μg/kg) and db-cyclic AMP (50 mg/kg) for seven consecutive days post-SCI (**o**–**q**) increased the number of Isolectin-IB4 positive immune cells (*red*) that were immunoreactive for Arginase-1 (*green*) within the lesion site. The nuclei of the cells have been counterstained with Hoechst (*blue*). *Scale bar* = 40 μm. **r** Quantitative assessment of the numbers of Isolectin-IB4+ cells immunoreactive for Arginase-1 showed a significant increase with db-cyclic AMP and IL-4 after SCI. Statistical significance indicated at ^###^ *p* < 0.001 versus SCI-only controls

To investigate whether M1 to M2a phenotypic conversion of microglia could be accomplished in vivo using cyclic AMP elevation and IL-4 supplementation, their concurrent delivery was investigated after contusive spinal cord injury (SCI) in two different experimental paradigms. First, in a rodent model, adult Lewis rats were subjected to injury and the co-administration of IL-4 and db-cyclic AMP systemically during the acute phase (within 15 min) of SCI. Using the prototypical M2a marker Arginase-1, at 24 h after SCI an increase in Arginase-1 expression was identified in the total spinal cord tissue homogenates of the lesion site compared to naïve controls by immunoblotting (5.9-fold increase compared to naïve, *p* < 0.05 Fig. 4a, b); indeed, an increase in both M1 and M2 microglia and macrophage populations has been reported previously in the acute phase of SCI [10]. The use of M1 to M2a phenotype conversion with db-cyclic AMP and IL-4 resulted in a significant enhancement of Arginase-1 production at 24 h after SCI (11.1-fold increase compared to naïve, *p* < 0.01 and *p* < 0.05 versus SCI; Fig. 4a, b). Histological examination of tissue sections from the injury epicenter showed that, compared to SCI controls (Fig. 4c, d), the combination of db-cyclic AMP and IL-4 after SCI produced a marked reduction in numbers of Iba1^+^ (Fig. 4e) and ED1^+^ (Fig. 4f) microglia and macrophages within the lesion site. Co-staining of ED1^+^ microglia or macrophages with Arginase-1 revealed that at 24 h after SCI alone, only a small fraction of ED1^+^ cells were immunoreactive for Arginase-1 (Fig. 4d–f; white arrows). In contrast, when M2a phenotype conversion with db-cyclic AMP and IL-4 was used acutely after SCI, almost all the ED1^+^ microglia and macrophages were immunoreactive for Arginase-1 (Fig. 4h–j; white arrows), indicating that they were of the M2a phenotype. To assess the effects of M2 microglia and macrophage conversion on the pathological processes of injury, we assessed at 24 h the generation of total free ROS/RNS radicals within the lesion site by employing the fluorogenic probe dichlorodihydrofluorescin DiOxyQ (DCFH-DiOxyQ), which is a specific ROS/RNS probe [23]. Compared to SCI controls, the combination of db-cyclic AMP and IL-4 to induce M1 to M2a phenotypic conversion produced a significant reduction in the amount of free radicals generated within the injured spinal cord (57.6 % reduction, *p* < 0.05; Fig. 4k).

In subsequent work, we employed a murine model of moderate SCI using c57/Bl6 mice. These mice received either SCI alone or SCI with the co-administration of IL-4 and db-cyclic AMP acutely post-SCI and then daily for 7 days. Immunohistochemical analysis of spinal cord tissue at the lesion site at 7 days post-SCI showed that compared to SCI controls (Fig. 4l–n), a significant increase in the number of Isolectin-IB4^+^ microglia and macrophages immunoreactive for Arginase-1 occurred with the concurrent delivery of IL-4 and db-cyclic AMP (Fig. 4o–q). Arginase-1 immunoreactivity at the center of the lesion appeared largely restricted to Isolectin-IB4^+^ cells. Quantitative assessment of the number of Isolectin-IB4^+^ microglia and macrophages that co-stained with Arginase-1 after SCI showed a significant increase with IL-4 and db-cyclic AMP over SCI controls (3.0-fold increase; *p* < 0.001; Fig. 4r).

## Discussion

Trauma to the CNS produces an intense and chronic reaction to the injury by resident microglia and infiltrating macrophages that perplexingly when abrogated can lead to tissue preservation and a reduction in neurological deficits [24] or, when specifically enhanced, can produce repair [25]. Recent work has attributed these diverse activities to the heterogeneity of the microglia and macrophage responses to injury, with the presence of distinct immunophenotypical populations of these cells providing unique functional roles within the injury environment [10, 26]. These phenotypes are broadly defined into two forms based upon gene profiling and proteomics, the classically activated M1 form and the alternatively activated M2 form, though it is clear that further work remains in the molecular identification of distinct microglial cell populations in vivo that possess different behavioral characteristics.

Though our understanding of the complexity of the microglia and macrophage responses following injury requires further experimentation to elucidate how these different populations alter the injury environment towards one of tissue damage or repair, it does appear based upon temporal population dynamics within the lesion milieu that the conversion of one phenotype to another is possible [10, 27, 28] and that factors present within the injury microenvironment strongly regulate the microglia and macrophage phenotypic responses and function [9, 29, 30]. The dominating role of signals from the injury milieu in driving the M1 phenotype was recently demonstrated in studies by Kigerl et al., [10] where they showed that M2-converted macrophages, induced with IL-4, while retaining their phenotype when microinjected into the intact spinal cord, were converted into the M1 state when injected into the injured spinal cord. Indeed, while both M1 and M2 microglia and macrophages are present within the acutely injured spinal cord, the M2 response is short-lived, dissipating within 3–7 days after injury [10], pointing to an environment supportive of only the M1 phenotype [31].

With the injured CNS showing concurrently a dramatic and chronic reduction in levels of the important secondary messenger, cyclic AMP [32], and based upon the important role that cyclic AMP plays in maintaining both microglia and monocyte homeostasis to prevent M1 activation [15, 33], we hypothesized that the elevation of cyclic AMP would be a pre-requisite to pro-M2 anti-inflammatory cytokine stimulation for a persistent M2 phenotypical conversion from an M1 activated state.

As shown in the present report, the combination of cyclic AMP elevation and stimulation with IL-4, but neither agent alone at the doses employed, was able to convert LPS-activated, M1 microglia to an M2a phenotype as characterized by a number of specific M1 and M2a markers. Though further dose-finding studies with the prototypical M2 inducer IL-4 alone [34–36] may allow the determination of a concentration of IL-4 that can achieve a significant induction of M2 markers in these cell culture systems, it is clear from this work that cyclic AMP provides a powerful synergism with IL-4 to induce M1 to M2a microglial conversion where individually these agents failed to at doses that are physiologically relevant. We and others have shown that cyclic AMP-elevating agents, including adenylyl cyclase activators, phosphodiesterase inhibitors, and synthetic analogs can retard the classical (M1) activation of microglia or macrophages largely through antagonism of the master transcriptional regulator of inflammation, nuclear factor kappa B (NF-κB; [11, 33]). In addition, cyclic AMP can also synergistically act with IL-4 to induce the expression of M2-regulated genes, such as Arginase-1, by activating the transcription factor CCAAT/enhancer-binding protein (C/EBP) [37]; thus, both of these mechanisms are likely important for the interplay of these signals in promoting persistent M1 to M2 conversion. According to recent sub-type classification of the M2 phenotype by immunocytochemical markers [9], the cyclic AMP and IL-4 converted microglia retain the M2a form; the substitution of IL-4 with other anti-inflammatory cytokines, such as IL-10 or TGF-β, in conjunction with cyclic AMP failed to recapitulate this M1 to M2a microglia conversion (data not shown), while IL-13 produced an analogous response to IL-4 with cyclic AMP (unpublished data). Functionally, the M2a conversion of M1 microglia with cyclic AMP and IL-4 significantly abated their production of pro-inflammatory cytokines (TNF-α) and chemokines (IP-10) and oxidative molecules (ROS/RNS), while increasing their phagocytic function. The production of pro-inflammatory cytokines and oxidative metabolites are key effectors of M1 microglia in exacerbating tissue damage after CNS injury [2], while for M2 microglia, the down-regulation of these inflammatory factors coupled with enhanced phagocytosis and ECM remodeling are important for their reparative actions [4].

While in the current study, microglial cells treated with LPS did not exhibit Arginase-1 expression, other work has shown that LPS can increase Arginase-1 within microglia in vitro [38] and in vivo [39, 40]. However, in each of these cases, there was the presence of additional stimuli or environmental factors that could have altered the response of microglia to LPS. In work by Lisi and colleagues [38], LPS stimulation occurred in the presence of conditioned media from C6 Glioma cells, media containing growth factors, and cytokines that could have altered the response to LPS leading to Arginase-1 expression. Similarly, in a study by Zhang and co-workers [40], involving a model of Endotoxin-induced uveitis (EIU), the break-down of the blood-ocular barrier and the infiltration of leukocytes in the retina following administration of LPS may have led to indirect effects on the microglia from secreted factors from leukocytes [41] or other glial cells (Muller glia). Secreted factors from these cells could have altered the response of the microglia to LPS in their production of Arginase-1. This environmental difference could also be a reason for the observed induction of Arginase-1 in cortical microglia following intraparenchymal injection of LPS in the brain [40]. Examining how LPS-induced changes in Arginase-1 expression by microglia may be altered by the presence of cytokines, growth factors, and other cell types would shed light on how the signaling pathways downstream of these factors are integrated in regulating microglial cell phenotype.

Through the use of PKA- and EPAC-selective cyclic AMP analogs, we demonstrated the importance of PKA, but not EPAC, in facilitating M1 to M2a microglia conversion in the presence of IL-4. Studies have shown that in LPS activated RAW 264.7 macrophages, the effects of cyclic AMP in reducing pro-inflammatory cytokine production, a hallmark of the M1 phenotype, is mediated through PKA [42]. Conversely, other work employing the same macrophage cell model has highlighted a role for Epac1-mediated Rap1/NFκB signaling in facilitating LPS-induced inflammatory responses [43]. While the use of EPAC and PKA agonists in the current study at concentrations previously shown to provide sufficient activation of the target in vitro [44] demonstrate the importance of PKA and not EPAC in M1 to M2a conversion and are in line with previous reports [43] that suggest the involvement of PKA and not EPAC as being the downstream mediator of M1 to M2 conversion; the use of multiple, higher doses of the EPAC agonists would be needed to completely rule out a role of EPAC in this process. Nevertheless, if demonstrated, it would indicate that selective PKA activation rather than broad cyclic AMP-elevating approaches will be required for persistent M2 conversion to avoid putative antagonistic effects of EPAC activation.

Not only could M1 to M2a conversion be obtained phenotypically and functionally with combined cyclic AMP and IL-4 in vitro in the BV2 microglial cell line and primary microglia but also in vivo in models of SCI in rat and mouse. While only a small population of macrosialin-positive microglia and macrophages within the injured spinal cord at 24 h after SCI co-expressed the M2a marker Arginase-1, following the systemic administration of cyclic AMP and IL-4, almost all macrosialin immunoreactive microglia and macrophages were also Arginase-1 positive. In addition to promoting M1 to M2a conversion, this treatment also reduced the total numbers of macrosialin and iba-1 positive microglia and macrophages within the injured spinal cord. This reduction could be produced by an inhibitory effect of cyclic AMP on macrophage infiltration as observed previously with cyclic AMP-elevating approaches [20, 45]. A similar increase in Arginase-1 with cyclic AMP and IL-4 after SCI was observed also by immunoblotting, and the in vivo M1 to M2a conversion after SCI was accompanied by a significant reduction in the generation of ROS. In SCI mice treated with IL-4 and a cyclic AMP analog for 7 days after injury, a similar significant increase in Arginase-1^+^ M2a microglia and macrophages was observed with the combination of agents over injury-only controls, substantiating this approach as a means to promote M1 to M2a conversion in vivo after CNS injury as a putative therapeutic strategy for inducing tissue repair.

Although direct spinal tissue measurements of db-cAMP and IL-4 were not performed in this study, following SCI, there is acute blood-spinal cord-barrier (BSCB) disruption to macromolecules for a least 1 week [46]. Disruption of the BSCB during the 7-day administration period of the agents would thus permit systemically administered IL4 and db-cAMP to readily reach spinal tissue at the lesion site. Should longer administration windows be required for achieving an effective treatment with this approach following SCI, the examination of spinal cord penetrance would be important to ensure that adequate levels were present or an alternative, more direct route for delivery, or small molecule mimetics/activators of these pathways would be needed.

## Conclusions

**Fig. 5:**
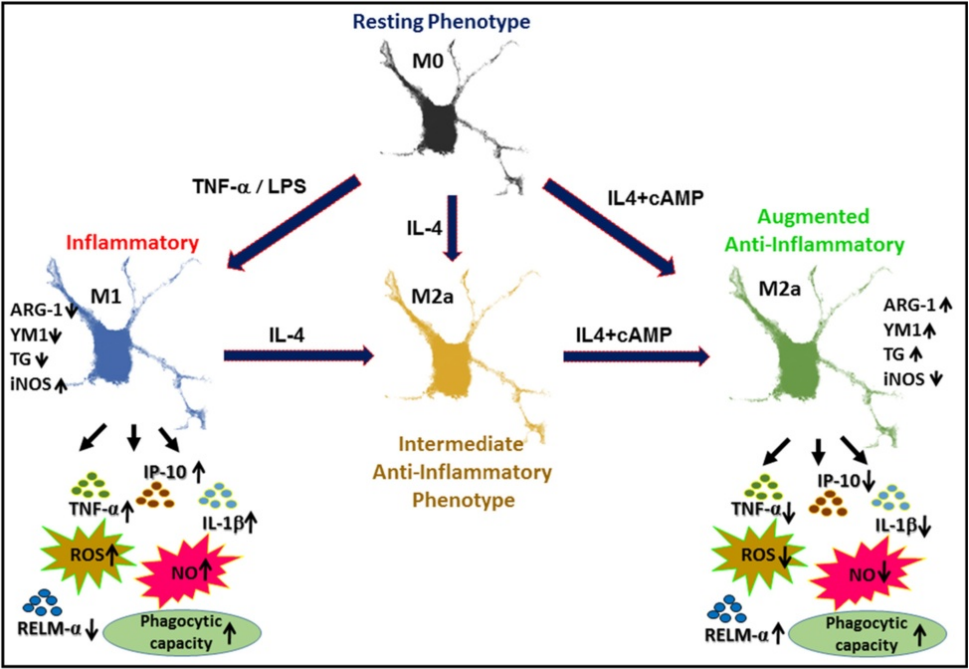
Synergistic immuno-modulatory effects of cyclic AMP and IL-4 in the M1 to M2a phenotypic conversion of microglia. Subjecting resting (M0) microglia to a pro-inflammatory stimulus, TNF-α or LPS, drives the cells to a classically activated (M1) phenotype. The M1 form displays high expression of cytokines and chemokines, such as TNF-α and IP-10, as well as iNOS and free radicles, ROS and RNS, that are key in cytotoxicity and tissue injury. In addition, the production of tissue reparative and remodeling enzymes, such as ARG-1, YM1, RELM-α, and TG, are suppressed. In contrast, the concurrent exposure of M0 or M1 microglia to IL-4 and cyclic AMP induces M0/M1 phenotype conversion to a robust reparative M2a form. The M2a phenotype exhibits strong expression of ARG-1, TG2, RELM-α, and YM1, an augmented phagocytic ability and the absence of pro-inflammatory cytokine and toxic reactive radical production that are associated with the M1 form. When M0- or M1-activated microglia are exposed to IL-4 alone, there is a transition to an intermediate M2a form. The M2a phenotype retains partial characteristics of M1 while acquiring some beneficial characteristics of the M2 phenotype, though exhibits impaired phagocytic function

These studies identify a novel method for M1 to M2a conversion that could hold great clinical promise as a therapeutic for CNS injury and disease in altering the microglia and macrophage responses from one of tissue injury to one of repair. Undertaking further in vivo work to optimize dosing of the agents and to determine the persistence of phenotypic conversion after CNS injury would pave the way for studies to examine the effectiveness of this new approach to provide anatomical protection, neurorepair, and functional benefit. It is also clear that further characterization of the synergistic mechanisms involved in M1 to M2 conversion and improved characterization of the relationship between the molecular profile of distinct microglia phenotypes and their behavioral responses is needed. As summarized in Fig. 5, the current work highlights for the first time the importance of cyclic AMP-PKA signaling in both antagonizing M1 microglia activation and inducing the M2a state in conjunction with anti-inflammatory cytokines, such as IL-4, to promote M1 to M2a phenotypical conversion.
